# Comprehensive Metabolite Profiling and Microbial Communities of *Doenjang* (Fermented Soy Paste) and *Ganjang* (Fermented Soy Sauce): A Comparative Study

**DOI:** 10.3390/foods10030641

**Published:** 2021-03-18

**Authors:** Da Hye Song, Byung Hee Chun, Sunmin Lee, Su Young Son, Chagam Koteswara Reddy, Ha In Mun, Che Ok Jeon, Choong Hwan Lee

**Affiliations:** 1 Department of Bioscience and Biotechnology, Konkuk University, Seoul 05029, Korea; thddl1213@naver.com (D.H.S.); duly123@naver.com (S.L.); syson119@naver.com (S.Y.S.); koteswarreddychagam@gmail.com (C.K.R.); nun6475@naver.com (H.I.M.); 2 Department of Life Science, Chung-Ang University, Seoul 06974, Korea; cbh0813@naver.com; 3 Department of Systems Biotechnology, Konkuk University, Seoul 05029, Korea; 4 Research Institute for Bioactive-Metabolome Network, Konkuk University, Seoul 05029, Korea

**Keywords:** fermented soybean paste, metabolomics, microbial community

## Abstract

*Doenjang* and *ganjang* are secondary fermented soybean products from *meju* (primary fermented product) following a complex fermentation process that separates the products into solid (*doenjang*) and liquid (*ganjang*) states. We performed a comparative study on gas chromatography mass spectrometry-(GC-MS) and liquid chromatography mass spectrometry-(LC-MS) based metabolite profiling with fungal and bacterial microbial community analysis of *doenjang* and *ganjang* during fermentation. Metabolite profiling and microbial community data showed distinct patterns, depending on the fermentation process. The relative levels of metabolic patterns were similar and most of the microorganisms produced halophilic or halotolerant microbes during the fermentation period in *doenjang* and *ganjang*. In the *doenjang* end products, isoflavones, soyasaponins, and amino acids were largely distributed and *Debaryomyces* and *Staphylococcus* were dominant, whereas the biogenic amine and phenylpropanoid contents were highly distributed in the *ganjang* end products, with higher levels of *Meyerozyma* and *Tetragenococcus*. Our results demonstrate that the quality of *doenjang* and *ganjang* is predominantly influenced by the microbiome and by metabolite changes during fermentation. Moreover, the present study provides a platform for comparing samples in different states.

## 1. Introduction

Fermentation is one of the oldest methods of food preservation and fermented dairy products and their microbial and functional characteristics have been intensively studied. Fermented soybean products are processed into a wide variety of foods, including soybean sprouts, soymilk, tofu, *doenjang*, *cheonggukjang*, *natto*, and *ganjang*. Fermented soybean foods have garnered considerable attention because of their excellent nutritional value, and they are recognized as functional foods. *Doenjang* and *ganjang* are traditional fermented soybean foods that have been reported to exert a variety of beneficial health effects such as anticancer, anti-cholesterol, anti-diabetic, antioxidant, anti-microbial, antigenotoxic, and anti-inflammatory effects [1]. *Doenjang* and *ganjang* are produced from *meju* following a complex fermentation process. To produce *meju* (primary fermentation product), soybeans are pretreated by steaming, shaping, and drying, followed by fermentation using microorganisms. Subsequently, the process of brining (i.e., adding salt water to *meju*) proceeds. The separated solid and liquid states are further aged for several months (secondary fermentation) to produce *doenjang* (fermented soy paste) and *ganjang* (fermented soy sauce) [2]. During the complex soybean fermentation process, various biochemical transformations (enzymatic hydrolysates such as amino acids and peptides produced during fermentation) occurred due to various microorganisms, which is known to improve the properties of the food [2]. *Meju*, *doenjang*, and *ganjang* represent complex microbial ecosystems comprising bacteria as well as fungi, which are responsible for the hydrolysis of the major ingredients, including proteins, lipids, carbohydrates, and flavonoid glycosides, during fermentation. Furthermore, a variety of metabolites produced by the microorganisms, such as amino acids, organic acids, active metabolites, and aglycones, contribute to the nutritional value of *doenjang* and *ganjang* [3]. Various microorganisms are involved in these fermentation processes, and their unique flavor originates from microbially decomposed soybean proteins by microbial action during the overall fermentation process in *doenjang* and *ganjang* [4]. The untargeted metabolomic studies involving *doenjang* and *ganjang* with raw material may unravel the biochemical aspects of associated nutritional and functional changes during fermentation and may help identify quality biomarkers for standardization. Metabolomics is a useful approach for analyzing metabolite changes during the fermentation of fermented foods such as other soybean fermented foods [5,6], and metabolomic methods generate large complex datasets and require advanced statistical and bioinformatics tools to assist in data interpretation [7]. Recently, the identification of the microbial composition of fermented foods, including *doenjang*, accelerated the development of rapid and accurate analytical techniques [8], and comparative studies on the microbial communities of *doenjang* and *ganjang* have been conducted [9]. Although *doenjang* and *ganjang* are made from the same raw material, it is difficult to compare the metabolite content of *doenjang* and *ganjang* in different states. Therefore, comparative metabolite changes during the aging of different fermentation products have not yet been reported. We used multivariate statistical analyses presented in two ways to provide a platform for comparing *doenjang* and *ganjang* in different states. In addition, non-targeted metabolomics may be useful for comparing and analyzing metabolites in *doenjang* and *ganjang* produced from the same raw material.

## 2. Materials and Methods

### 2.1. Chemicals and Reagents

Analytical-grade methanol, acetonitrile, and water were purchased from Thermo Fisher Scientific (Pittsburgh, PA, USA). Reagent-grade chemicals, including methoxyamine hydrochloride, pyridine, and N-methyl-N-(trimethylsilyl)-trifluoroacetamide (MSTFA), were obtained from Sigma-Aldrich (St. Louis, MO, USA).

### 2.2. Materials

Fifteen *meju* bricks (i.e., fermented soybean bricks of approximately 1.6 kg each), solar salt, and minor ingredients, including dried hot red pepper and charcoal lumps (approximately 12 g each), were purchased from a *meju* manufacturing unit located in Jeollabukdo area, South Korea.

### 2.3. Preparation of Doenjang and Ganjang

**Figure 1:**
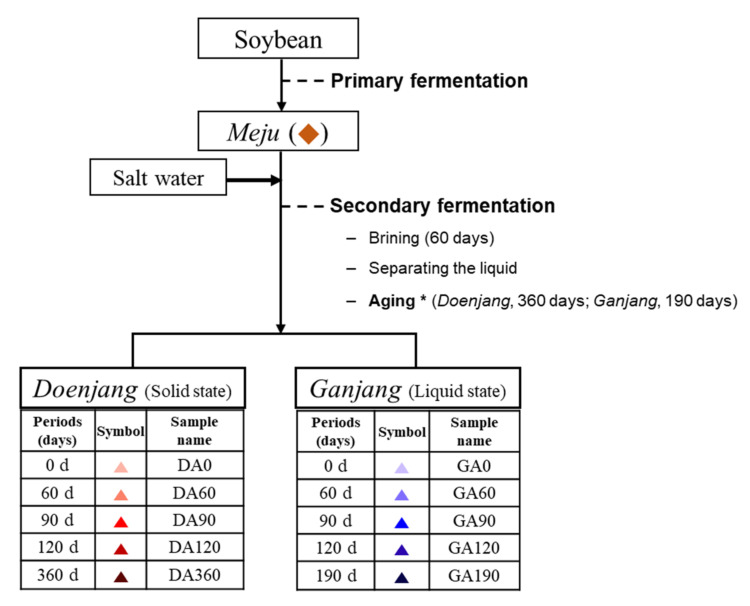
Overview of the processes involved in the production of *doenjang* and from *meju* (primary fermented product). Different processes indicated as: *meju* (◆), *doenjang* aging (0 d: ▲; 60 d: ▲; 90 d: ▲; 120 d: ▲; 360 d: ▲), *ganjang* aging (0 d: ▲; 60 d: ▲; 90 d: ▲; 120 d: ▲; 190 d: ▲ ). * The period for making final products.

*Doenjang* and *ganjang* samples were prepared in triplicate according to a traditional manufacturing method (Figure 1) [10]. Briefly, four fermented *meju* (primary fermented product) bricks, two dried hot red peppers, and one lump of charcoal were mixed with 20 L solar saline (18%, *w/v*) in a Korean porcelain pot. The mixture was then subjected to a brining process of storing at room temperature (about 25 °C) for 60 days. Subsequently, the mixtures were separated into the solid state (*doenjang*) and liquid state (*ganjang*), and the samples were stored in two different smaller porcelain pots, according to traditional methods. This time point was designated as day 0 of aging. To prepare the *doenjang* samples, the solid *meju* was thoroughly mashed and placed compactly in the porcelain pots. The pots containing *doenjang* and *ganjang* were stored at room temperature for 360 and 190 days, respectively, and *doenjang* and *ganjang* samples were periodically collected.

For microbial community analyses of *doenjang*, *doenjang* samples (1 g) collected from the triplicate porcelain pots were pooled, thoroughly mixed, and stored at −80 °C until analysis. For metabolite analyses, each *doenjang* sample (1 g, un-pooled) was separately freeze-dried and was stored at −80 °C. For microbial community analyses of *ganjang*, each sample (2 mL) was centrifuged at 18,894× *g* and 4 °C for 10 min, and the pellets of three replicates were pooled and stored at –80 °C. The supernatants were separately freeze-dried (un-pooled) and stored at −80 °C for metabolite analysis. In addition, *meju* samples used to prepare the *doenjang* and *ganjang* mixtures were also processed for microbial community and metabolite analyses.

### 2.4. Bacterial and Fungal Community Analysis

Total genomic DNA was extracted from pooled samples using a FastDNA Spin kit (MPbio, Solon, OH., USA) according to the manufacturer’s instructions. Sequences of the V3–V4 region of the bacterial 16S rRNA gene and of the internal transcribed spacer (ITS) 2 region of the fungal rRNA gene were PCR-amplified using the two universal primer sets 341F (5′-adaptor-CCT ACG GGN GGC WGC AG-3′) plus 805R (5′-adaptor-GAC TAC HVG GGT ATC TAA TCC-3′) and 3271-ITS2F (5′-adaptor-CAR CAA YGG ATC TCT TGG-′3) plus 3271-ITS2R (5′-adaptor-GAT ATG CTT AAG TTC AGC GGG T-3′), respectively, and were sequenced using an Illumina MiSeq platform (Roche, Germany) at Macrogen (Seoul Korea), as described previously [11]. The resulting sequencing reads were sorted into *doenjang*, *ganjang*, and *meju* samples according to their barcode sequences, and the barcode and adapter sequences were removed using Scythe software (v.0.994, https://github.com/vsbuffalo/scythe, accessed on 13 February 2020). Bacterial and fungal communities were analyzed using the Qiime2 plugin software package (v.2020.2, https://qiime2.org/, accessed on 13 February 2020), according to a previously described method [12]. Microbial diversity indices, including operational taxonomic units (OTU), Chao1 richness, and the Shannon–Weaver index, were calculated using the Qiime2 pipeline.

### 2.5. Gas Chromatography Time-of-Flight Mass Spectrometry (GC-TOF-MS) Analysis

Each dried sample extract was subjected to two derivatization reactions, following the method described by Lee et al. [2]. All the derivatized samples were filtered using a Millex GP 0.22-µm filter (Merck Millipore, Billerica, MA, USA) before analysis. GC-TOF-MS analysis was performed using an Agilent 7890 gas chromatography system, coupled with an Agilent 7693 autosampler (Agilent, Santa Clara, CA, USA) and a Pegasus high-throughput (HT)-TOF-MS program (Leco Corp., St. Joseph, MI, USA). Metabolites were separated using an Rtx-5MS column (30 m × 0.25 mm i.d.; 0.25 µm particle size; Restek Corp. Bellefonte, PA, USA), which was used with helium as a carrier gas at a 1.5 mL/min flow rate. The GC oven temperature was 75 °C for 2 min, which was increased to 300 °C for 15 min at a rate of 15 °C/min. The front inlet and sp temperatures were maintained at 250 °C and 240 °C, respectively. One microliter of each sample was injected at a split ratio of 10:1, *v/v*, with mass spectra recorded over a range of 50–1000 *m/z*. The detector voltage was 1660 V. We used three technical replicates of each sample, and analyses were performed in random order to reduce bias.

### 2.6. Ultrahigh Performance Liquid Chromatography-Orbitrap-Mass Spectrometry/Mass Spectrometry (UHPLC-orbitrap-MS/MS) Analysis

A UHPLC system equipped with a Vanquish binary pump H system (Thermo Fisher Scientific, Waltham, MA, USA) was united with an auto-sampler and column compartment. A Phenomenex KINETEX^®^ C18 column (100 mm × 2.1 mm, 1.7 µm particle size; Torrance, CA, USA) was used to chromatographic separation. The injection volume was 5 µL. The column temperature was set to 40 °C, and the flow rate was 0.3 mL/min. The mobile phase consisted of 0.1% *v/v* formic acid in water (A) and acetonitrile (B). The gradient parameters were configured as follows: Five percent solvent B was maintained initially for 1 min, followed by a linear increase to 100% solvent B over 9 min and then sustained at 100% solvent B for 1 min, with a gradual decrease to 5% solvent B over 3 min. The total run time was 14 min. The MS data were collected in a range of 100–1500 *m/z* (in negative- and positive-ion mode) using an Orbitrap Velos Pro system (Thermo Fisher Scientific, Waltham, MA, USA) coupled with an ion-trap mass spectrometer coupled with a heated electrospray ionization (HESI-II) probe. The probe heater and capillary temperatures were set to 300 °C and 350 °C, respectively. The capillary voltage was set to 2.5 kV in negative mode (positive mode 3.7 kV).

### 2.7. Data Processing and Multivariate Statistical Analysis

GC-TOF-MS and UHPLC-Orbitrap-MS/MS raw data files were converted according to a method described by Lee et al. [2]. The resulting data, including sample name and peak area information as variables, were processed using SIMCA-P+ 12.0 (Umetrics, Umea, Sweden) for multivariate statistical analyses. Data sets were subjected to principal component analysis (PCA and 3D-PCA) and partial least squares discriminant analysis (PLS-DA) with group-specific bias. The discriminant metabolites were selected based on variable importance in projection variable importance in projection (VIP) values (>1.0). In addition, we used information on metabolites that were tentatively identified using various data, including mass fragment patterns, retention times, and mass spectra of standard compounds, under the same conditions as those described in published studies and commercial databases such as the National Institutes of Standards and Technology (NIST) Library (v.2.0, 2011, FairCom, Gaithersburg, MD, USA), The Dictionary of Natural Products (v.16:2, 2007, Chapman and Hall, USA), Wiley 8,and the Human Metabolome Database (HMDB; http://www.hmdb.ca/, accessed on 13 February 2020). Significance was tested via one-way ANOVA using Statistica (version 7.0, StatSoft Inc., Tulsa, OK, USA). Pearson’s correlation coefficient between metabolites and the corresponding phenotype was determined using predictive analytics software (PASW) Statistics 18 software (SPSS Inc., Chicago, IL, USA).

### 2.8. Bioactivity Assay Analysis

Bioactivities of the *meju*, *doenjang*, and *ganjang* samples in 80% aqueous methanol at a final concentration of 20 mg/mL were assessed following the methods reported by Lee et al. [13] and Son et al. [14]. 2,2′-Azino-bis (3-ethylbenzothiazoline-6-sulfonic acid) (ABTS) radical-scavenging activity assays and ferric reducing antioxidant power (FRAP) assays were performed to assess the in vitro antioxidant activity. The antioxidant results are represented as Trolox equivalent antioxidant capacity (TEAC) concentrations (mM). Total phenolic content (TPC) and total flavonoid content (TFC) were measured. The results of the TPC and TFC assays are expressed as the gallic acid equivalent activity (μg/mL) and naringin equivalent activity (μg/mL), respectively.

## 3. Results and Discussion

### 3.1. Metabolite Profiling of Doenjang (Solid) and Ganjang (Liquid)

**Figure 2:**
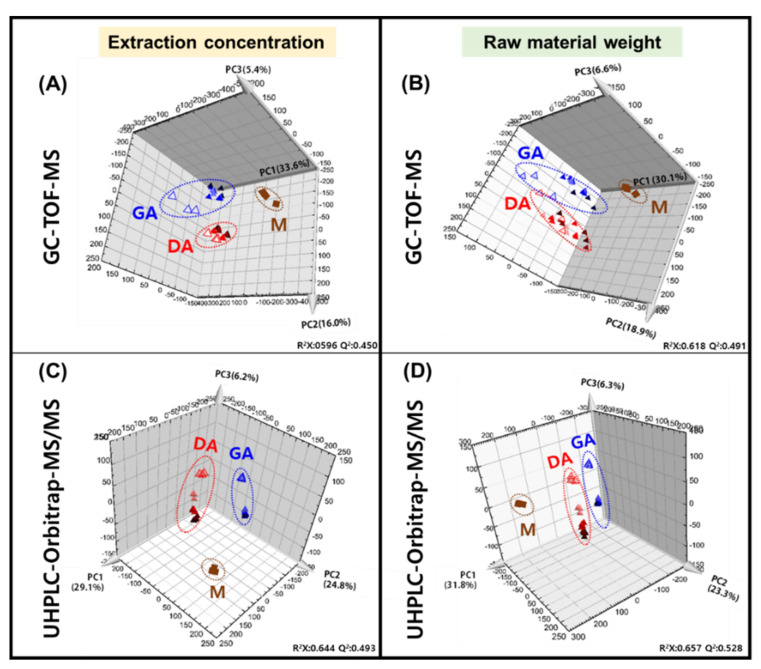
PCA score plots based on concentrations, as derived from Gas Chromatography Time-of-Flight Mass Spectrometry (GC-TOF-MS) (**A**) and Ultrahigh Performance Liquid Chromatography-Orbitrap-Mass Spectrometry/Mass Spectrometry (UHPLC-Orbitrap-MS/MS) (**C**). Principal component analysis (PCA) score plots normalized to the raw material weight, as derived from GC-TOF-MS (**B**) and UHPLC-Orbitrap-MS/MS (**D**). Different processes are symbolized as *meju* (raw material: ◆), *doenjang* aging (0 d: ▲ ; 60 d: ▲; 90 d: ▲; 120 d: ▲; 360 d: ▲), and *ganjang* aging (0 d: ▲; 60 d: ▲; 90 d: ▲; 120 d: ▲; 190 d: ▲).

*Doenjang* and *ganjang* are the traditional fermented products derived from *meju* (primary fermented product) with secondary fermentation such as brining, separating the liquid, and aging. The fermentation process of *doenjang* and *ganjang* from *meju* is shown in Figure 1. The biochemical processes underlying the fermentation of metabolites and the changes during the fermentation of *doenjang* and *ganjang* have not been studied extensively. We performed metabolite profiling of *meju* (M), *doenjang* aging (DA), and *ganjang* aging (GA) using GC-TOF-MS and UHPLC-Orbitrap-MS/MS. Multivariate statistical analyses, principal component analysis (PCA), and partial least squares discriminant analysis (PLS-DA) were used to confirm differences in the metabolites of *meju*, DA, and GA. PCA is a dimensional reduction technique to visualize multiple-scaled metabolomics data, and the unsupervised classification technique is used to identify differences among samples and identify variables that contribute to those differences [13]. PLS-DA was performed to select the discriminant metabolites added to the observed variance (Figure S1). The PLS-DA score plot revealed a pattern similar to that of the PCA. In the multivariate analysis results, the statistical variants were indicated using R^2^X and R^2^Y, representing the total sum of squares; Q^2^ is the fraction of total variation for the X and Y components. Each of these variants is marked at the bottom of the PCA and PLS-DA plots (Figure 2 and Figure S1). Concentrations (mg/mL) are shown as base data and data normalized to raw material weight (mg = μL). The variance of each PC of the concentration-based three-dimensional (3D) PCA score plots were PC1 (33.6%), PC2 (16.0%), and PC3 (5.4%) according to GC-TOF-MS (Figure 2A) and PC1 (29.1%), PC2 (24.8%), and PC3 (6.2%) in the UHPLC-Orbitrap-MS/MS analysis (Figure 2C). The raw material weight-based three-dimensional (3D) PCA score plots revealed PC1 (30.1%), PC2 (18.9%), and PC3 (6.6%) in the GC-TOF-MS (Figure 2B) and PC1 (31.8%), PC2 (23.3%), and PC3 (6.3%) in the UHPLC-Orbitrap-MS/MS (Figure 2D). The GC-TOF-MS and UHPLC-Orbitrap-MS/MS analyses showed a similar tendency in that the fermentation processes were clustered in each process, according to the two-way data. In the proposed four PCAs, *meju* was separated from DA and GA by PC1 and DA and GA were each separated by PC2 and PC3. The variables were selected from concentration-based data and normalized to the raw material weight (Figure S1). Metabolites that contributed to sample separation were selected based on their VIP values (>1.0). In total, 64 metabolites, including 29 primary metabolites and 34 secondary metabolites, were tentatively identified (Tables S1 and S2). The GC-TOF-MS data showed primary metabolites, including amino acids (1–14), fatty acids (15–16), organic acids (17–20), and sugar derivatives (21–29). Dipeptides (37–40), biogenic amines (41–43), phenylpropanoids (44), flavonoids (45–57), soyasaponins (58–65), and lipids (66–70) were identified from the UHPLC-Orbitrap-MS/MS data. The metabolites were tentatively identified using their mass fragment patterns and retention times in comparison with those of standard compounds using the NIST database and an in-house library.

**Figure 3:**
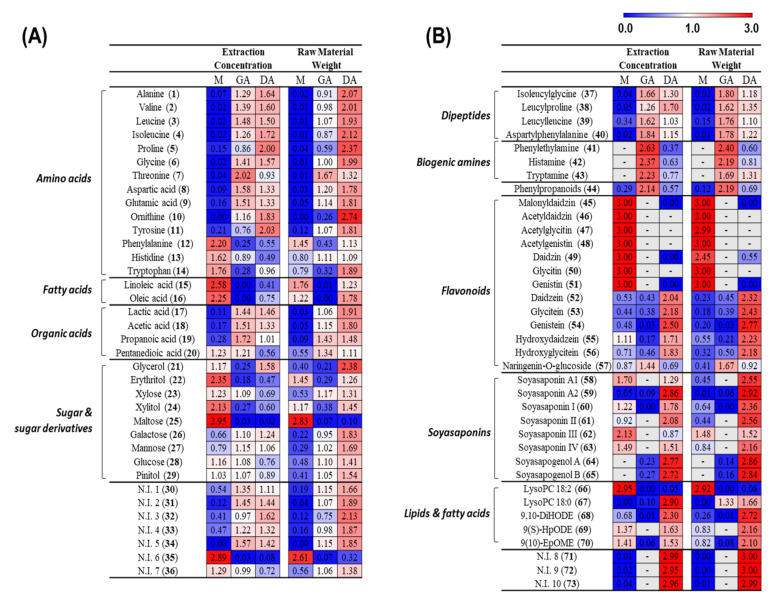
Heat map derived from GC-TOF-MS (**A**) and UHPLC-Orbitrap-MS/MS (**B**). A heat map includes data based on extract concentration and normalized raw material weight. The values represent fold changes by an average peak area of each metabolite (Variable importance in projection (VIP) > 1.0).

A GC-TOF-MS heat map (Figure 3A) demonstrated that the metabolite patterns differed significantly different between concentration (mg/mL) data and raw material weight (mg = µL) data. The concentration data showed a higher content of amino acids and organic acids in GA and DA than in *meju*, whereas the normalized data showed a trend of high content only in DA. In addition, sugar and sugar derivatives showed higher concentrations in *meju*, but the sugar content was higher in DA after normalizing the raw material weight. However, the content of secondary metabolites according the UHPLC-Orbitrap-MS/MS heat map trend was similar to the concentration data and raw material weight data (Figure 3B). Fatty acids, isoflavones, and soyasaponins are known to be the major compounds in soybean fermented foods [15,16]. These metabolites were abundant in *meju* and DA. Interestingly, dipeptide and biogenic amines were abundant in GA. As shown in Figure 2 and Figure 3, this trend changed after the conversion based on the raw material weight from the concentration data. In previous studies, a comparison of bacterial communities in *doenjang* and *ganjang* was used as a standard unit (g = mL) [4]. Therefore, we selected the raw material weight data to compare the metabolite components of aged *doenjang* and aged *ganjang* in different states.

### 3.2. Comparison of Metabolite and Microbial Changes during Doenjang and Ganjang Fermentation

#### 3.2.1. Metabolite Change

**Figure 4:**
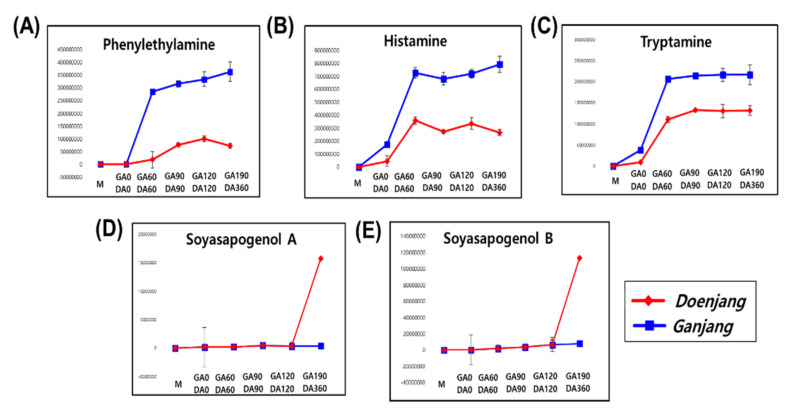
Profiles of biogenic amines (phenylethylamine, (**A**); histamine, (**B**); tryptamine, (**C**)), and soyasapogenols (soyasapogenol A, (**D**); soyasapogenol B, (**E**)). The *Y*-axis of the graph indicates peak areas. Differences were considered significant at *p*-value < 0.05. Data are presented as mean standard error from triplicates.

The changes in the relative concentrations of discriminant metabolites during the fermentation of *doenjang* and *ganjang* were comprehensively visualized in metabolic pathway network layouts (Figure S2). Each column shows the fold change value compared to *meju*. Both the DA (*doenjang* aging) and GA (*ganjang* aging) processes showed steeply increasing patterns of amino acid and organic acid concentrations. In previous studies, organic acids and amino acids increased during fermentation due to enzymes such as proteases and amylase produced by microorganisms [2]. These amino acid levels were substantially increased after separating the liquid in *doenjang* and *ganjang*. During the early fermentation period, amino acid concentrations rapidly increase and contribute to the sweet and umami taste of fermented soybean food products [17]. Alanine, glycine, and threonine are associated with a sweet taste, whereas glutamic acid and aspartic acid are associated with umami [18]. Additionally, amino acids, including leucine, valine, isoleucine, proline, tyrosine, and ornithine, which contribute to the bitter taste of soybean foods, exhibit a rapid increase after separating the liquid [8]. Fatty acid levels were lower in *ganjang*. Linoleic acid, oleic acid, 9,10-DiHODE, 9(S)-HpODE, and 9(10)-EpOME abundantly occurred in *doenjang.* Almost all sugar derivatives showed a rapid decrease, whereas mannose, pinitol, and glucose concentrations increased in both *doenjang* and *ganjang*. Sugars decrease because they are used as a substrate by acidic bacteria for organic acid fermentation [19]. Our results showed that despite differences in fermentation conditions, the trends of the primary metabolic pathways were similar in GA and DA. Isoflavones and soyasaponins are characteristic components of soybean products such as *meju*, *doenjang*, and *ganjang* [2,19]. Most isoflavones occur in various forms such as glycosides and aglycones, acetyl, and malonyl compounds [20,21]. Our results revealed that isoflavone glycosides, viz., malonyldaidzin, acetyldaidzin, acetylglycitin, acetylgenistin, daidzin, glycitin, and genistin, were decreased or eliminated, whereas isoflavone aglycones and hydroxy-aglycones, viz., daidzein, genistein, hydroxy-daidzein, and hydroxy-glycitein, were increased in DA. Previously, the aglycone and hydroxyisoflavone content was observed to increase during soybean food fermentation due to glucosidase-mediated glycoside hydrolysis [22]. The isoflavones aglycone, hydroxyisoflavone, and soyasapogenol (soyasaponin aglycone) were reported to increase during the fermentation period of *doenjang* [23]. We examined the conversion of secondary metabolites (isoflavone and soyasaponin) during fermentation and found that hydroxyisoflavone, aglycones, and soyasaponin increased in DA. Soyasapogenol A and B were not detected in *meju*; however, soyasapogenols occurred in DA and GA. In addition, biogenic amines such as histamine, tryptamine, and phenylethylamine were rapidly produced during DA and GA. The content change of the compounds produced during each aging period is presented in Figure 4. The pattern of rapidly increasing Soyasapogenol A-B was confirmed only in DA360. The three kinds of biogenic amines rapidly increased during the aging process; however, the variation of the *ganjang* was more dynamic. On the contrary, biogenic amine precursors (histidine, tryptophan, and phenylalanine) continued to increase until day 90 and then gradually decreased. Jung et al. [24] and Shukla et al. [25] observed biogenic amines in *doenjang* and *ganjang*. Biogenic amines are low-molecular-weight nitrogenous organic compounds that are produced via microbial decarboxylation of the corresponding amino acids and nitrogen compounds during food fermentation [24,26].

#### 3.2.2. Microbial Community Changes

**Figure 5:**
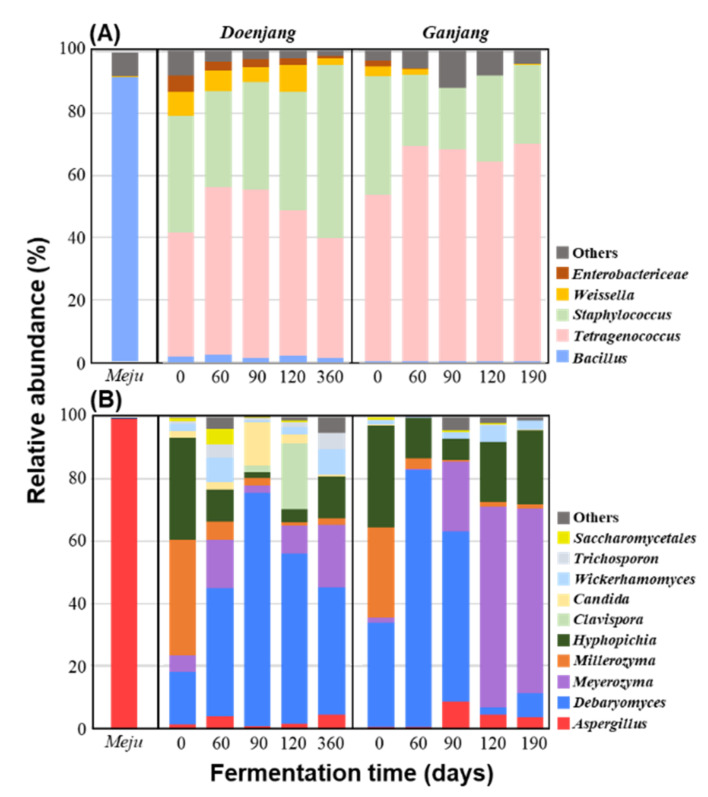
Taxonomic classification of the bacterial 16S rRNA and fungal ITS2 sequences at the genus level showing bacterial (**A**) and fungal (**B**) community changes during *doenjang* and *ganjang* fermentation with the bacterial and fungal communities of *meju*. “Others” comprise bacterial and fungal genera with less than 5.0% prevalence in all samples.

In total, 85,712 and 688,991 high-quality bacterial and fungal reads, respectively, were generated from the *doenjang*, *ganjang*, and *meju* samples, and their statistical diversity indices, including OTU, Chao1, and Shannon–Weaver, in each sample were calculated (Table S3). Bacterial and fungal diversities were slightly lower in *meju* than in *doenjang* and *ganjang*. Additional *doenjang* and *ganjang* microbes may have been introduced with other raw materials such as solar salts in addition to those present in *meju* [27]. Bacterial and fungal diversities during *doenjang* and *ganjang* fermentation fluctuated without a consistent decrease or increase, suggesting that microbial successions may have occurred during fermentation. The bacterial 16S rRNA and fungal ITS2 gene sequences of *doenjang*, *ganjang*, and *meju* samples were classified at the genus level to investigate microbial community changes during fermentation. Microbial communities in *doenjang* and *ganjang* were generally similar during fermentation (Figure 5). Bacterial community analyses revealed that the moderately halophilic or halotolerant genera *Tetragenococcus* and *Staphylococcus* were predominant in both *doenjang* and *ganjang* during the entire fermentation period, neither of which occurred in *meju* (Figure 5A). However, *Bacillus* was predominant in *meju* and was identified at very low levels in *doenjang* and *ganjang* during the entire fermentation period. *Bacillus* may not grow well in *doenjang* and *ganjang* under anaerobic and high-salt conditions, and its effects may not be significant. *Weissella* and *Enterobacteriaceae*, which are lactic acid bacteria and may be derived from *meju*, were identified at relatively high abundances in *doenjang*, suggesting that lactic acid bacteria may be important during *doenjang* fermentation. Previous studies reported that adding lactic acid bacteria improves the quality of *doenjang* [27]. In *ganjang*, lactic acid bacteria were identified at low abundances only during the early fermentation period, suggesting limited effects on *ganjang* fermentation. Fungal community analyses revealed that the genera *Debaryomyces*, *Meyerozyma*, *Millerozyma*, and *Hyphopichia*, which are halotolerant yeasts, were abundant during *doenjang* and *ganjang* fermentation (Figure 5B). *Aspergillus*, which was predominant in *meju*, occurred only at low abundances in *doenjang* and *ganjang* throughout the fermentation period, suggesting that this genus is not important for *doenjang* and *ganjang* fermentation. In *doenjang*, *Debaryomyces* was predominant during the entire fermentation period, and in *ganjang*, it was abundant only during early fermentation, whereas *Meyerozyma* was abundant during late fermentation. The genera *Clavispora*, *Candida*, *Wickerhamomyces*, and *Trichosporon* occurred at relatively low abundances during *doenjang* and *ganjang* fermentation. These results suggest that halophilic or halotolerant microbes such as *Tetragenococcus*, *Staphylococcus*, *Debaryomyces*, *Meyerozyma*, *Millerozyma*, and *Hyphopichia* may affect metabolite changes during DA and GA. During *doenjang* and *ganjang* fermentation, the abundances of *Bacillus* and *Aspergillus* that were predominantly present in *meju* became low, and instead fermentative and halophilc microbes such as *Tetragenococcus*, *Staphylococcus*, and *Debaryomyces* that originated probably from solar salts became dominant in *doenjang* and *ganjang* samples, which is thought to be because *doenjang* and *ganjang* are fermented under high salt and anaerobic conditions [12,27].

### 3.3. Comparative Analysis of the Doenjang and Ganjang End Products

**Figure 6:**
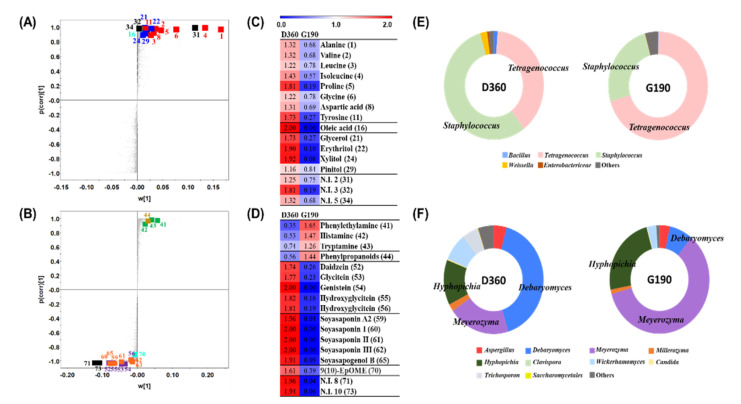
OPLS-DA S-plots derived from non-targeted metabolite profiling of *doenjang* and *ganjang* end products analyzed using GC-TOF-MS (**A**) and UHPLC-Orbitrap-MS/MS (**B**) (VIP > 1.5, *p* < 0.05). Metabolite compounds: amino acids (■), fatty acids (■), sugar and sugar derivatives (■), isoflavonoids (■), soyasaponins (■), biogenic amine (■), phenylpropanoids (■), and not identified (■). Heat map derived from GC-TOF-MS (**C**) and UHPLC-Orbitrap-MS/MS (**D**). The values represent fold changes by an average peak area of each metabolite. Relative abundances of bacteria (**E**) and fungi (**F**) present in *meju*, *doenjang* 360 d, and *ganjang* 190 d at the genus levels. “Others” comprise bacterial and fungal groups with <5% relative abundance in all samples.

In the pathway map, compounds that increased rapidly in DA and GA were not comparable; thus, the comparison was performed using each end product. The end products of *doenjang* after 360 days of fermentation (D360) and *ganjang* after 190 days (G190) were examined for discriminant metabolites using PCA and OPLS-DA models based on the GC-TOF-MS and UHPLC-Orbitrap-MS/MS data (Figure S3). The GC-TOF-MS and UHPLC-Orbitrap-MS/MS based on OPLS-DA score plots showed that D360 significantly differed from G190 along each OPLS1 (73.24% and 92.68%). The difference in metabolite content of the end product is suggested by the corresponding S-plots (Figure 6A,B) and heat maps (Figure 6C,D). The discriminant metabolites and their relative levels showed that amino acids, fatty acids, and sugar derivatives were primarily associated with D360. In addition, soybean-derived components such as isoflavones and soyasaponins, which are secondary metabolites, also showed high concentrations in D360. We suggest that this is because *doenjang* is a solid product, whereas *ganjang* is liquid with most of the soybean residue removed. The bioactivity of D360, which showed high concentrations of secondary metabolite-related compounds, was also significantly higher than that of G190 (Figure S4). Many studies reported that antioxidant activity is correlated with secondary metabolites, and major compounds of soybean such as flavonoids and isoflavonoids with a B-ring structure are well-known antioxidants [28]. In contrast, higher proportions of biogenic amines and phenylpropanoids were observed mainly in G190. Differences in the microbial distribution in the end products were revealed (Figure 6E,F). A comparison of the end products also showed that the relative distribution of fungi was more diverse than that of bacteria. *Tetragenococcus* is known to produce biogenic amines through the decarboxylation of amino acids or nitrogen compounds [11], and it was more abundant in *ganjang* than in *doenjang* (Figure 6E), which may have caused the high concentrations of biogenic amines in *ganjang* (Figure 6D). *Meyerozyma*, *Hyphopichia*, *Debaryomyces*, and *Aspergillus* were predominant in both D360 and G190, whereas *Wickerhamomyces* and *Trichosporon* were abundant only in D360. Lactic acid bacteria and *Debaryomyces* were more common in *doenjang* (Figure 5). These strains are known to produce *β*-glucosidase enzymes [29,30]. The enzyme affected the production of isoflavones and soya saponin aglycones by breaking down sugars. Isoflavone and soyasaponin aglycone concentrations were higher in D360 than in G190 (Figure 6C). The relative distribution of metabolites and microorganisms in the final products of *doenjang* and *ganjang* differed to some extent. The abovementioned species are generalists that have been reported to occur during fermentation [31,32,33,34,35]. We suggest that the enzymes produced by various microorganisms in D360 and G190 contribute to differences in the metabolome between products. In addition, we propose that differences in fermentation environments between the product environment are the main cause underlying metabolite and microbial distribution differences between D360 and G190. We compared *doenjang* and *ganjang* and demonstrated that environmental change is an important factor in fermentation.

## 4. Conclusions

In this study, we performed metabolite profiling and assessed the microbial communities of *meju, doenjang*, and *ganjang*. We compared samples in different states using two-way data based on extract concentrations and data normalized according to the raw material weight. The results of the identified metabolites revealed that metabolite trends changed in normalized data. Therefore, by comparing the metabolite pathway with the converted data, the metabolite pattern for each fermentation period of *doenjang* and *ganjang* was similar. In addition, the microbial communities of *meju*, DA, and GA contained predominant genera of halophilic or halotolerant microbes such as *Tetragenococcus, Staphylococcus, Debaryomyces*, *Meyerozyma*, *Millerozyma*, and *Hyphopichia*. Interestingly, when comparing the final products of *doenjang* and *ganjang*, most compounds in soybean fermentation products were found to be abundant in *doenjang*, whereas biogenic amines and phenylpropanoids were abundant only in *ganjang*. Furthermore, microbial community structures differed between *doenjang* and *ganjang*. Our results demonstrate that microbial community structures differ between the fermentation of different product states, and we conclude that these differences affect the metabolic components. Future research should be conducted to elucidate correlations between microorganisms and metabolites in *doenjang* and *ganjang*.

## Data Availability

The data presented in this study are available on request from the corresponding author.

## Supplementary Materials

The following are available online at https://www.mdpi.com/2304-8158/10/3/641/s1: Figure S1: PLS-DA score plots based on concentrations derived from GC-TOF-MS (**A**) and UHPLC-Orbitrap-MS/MS (**C**). PLS-DA score plots normalized to raw material weight derived from GC-TOF-MS (**B**) and UHPLC-Orbitrap-MS/MS (**D** Figure S2: Pathways of the relative levels of discriminant metabolites at different times of *doenjang* and *ganjang* as determined using the PLS-DA data sets based on raw material weight (VIP > 1.0) for GC-TOF-MS and UHPLC-Orbitrap-MS/MS analyses. The discriminant metabolites were further correlated with corresponding steps in the biosynthetic pathways adapted from the Kyoto Encyclopedia of Genes and Genomes database. The values indicate the log10-transformed fold changes based on the values of *meju*. Figure S3: PCA and OPLS-DA score plots derived from non-targeted metabolite profiling of *doenjang* and *ganjang* end products analyzed using GC-TOF-MS (**A**,**B**) and UHPLC-Orbitrap-MS/MS (**C**,**D**) (VIP > 1.5, *p* < 0.05). Figure S4: Antioxidant activity analysis using ABTS (**A**), FRAP (**B**), TPC (**C**), and TFC (**D**) of *doenjang* (D360), and *ganjang* (G190) end products. Different letters in the bar graph indicate significant differences as analyzed by ANOVA followed by Duncan’s new multiple range test. Table S1: Tentatively identified *meju* metabolites from different materials based on GC-TOF-MS analysis. Table S2: Tentatively identified *meju* metabolites from different materials based on UHPLC-Orbitrap-MS/MS analysis. Table S3: Bacterial and fungal sequence datasets derived from the *meju*, *doenjang*, and *ganjang* samples and statistical diversity analysis.

## References

[1] Yokota T., Hattori T., Ohishi H., Hasegawa K., Watanabe K. (1996). The effect of antioxidant-containing fraction from fermented soybean food on atherosclerosis development in cholesterol-fed rabbits. LWT-Food Sci. Technol..

[2] Lee S., Lee S., Singh D., Oh J.Y., Jeon E.J., Ryu H.S., Lee D.W., Kim B.S., Lee C.H. (2017). Comparative evaluation of microbial diversity and metabolite profiles in *doenjang*, a fermented soybean paste, during the two different industrial manufacturing processes. Food Chem..

[3] Jung J.Y., Lee S.H., Jeon C.O. (2014). Microbial community dynamics during fermentation of *doenjang*-*meju*, traditional Korean fermented soybean. Int. J. Food Microbiol..

[4] Cho K.-M., Seo W.-T. (2007). Bacterial diversity in a Korean traditional soybean fermented food (*doenjang* and *ganjang*) by 16S rRNA gene sequence analysis. Food Sci. Biotechnol..

[5] John K.M., Jung E.S., Lee S., Kim J.-S., Lee C.H. (2013). Primary and secondary metabolites variation of soybean contaminated with Aspergillus sojae. Food Res. Int..

[6] Baek J.G., Shim S.-M., Kwon D.Y., Choi H.-K., Lee C.H., Kim Y.-S. (2010). Metabolite profiling of Cheonggukjang, a fermented soybean paste, inoculated with various Bacillus strains during fermentation. Biosci. Biotechnol. Biochem..

[7] Gibbons H., O’Gorman A., Brennan L. (2015). Metabolomics as a tool in nutritional research. Curr. Opin. Lipidol..

[8] Kim M.J., Rhee H.S. (1990). Studies on the change of taste compounds during soy paste fermentation. Korean J. Soc. Food Sci..

[9] Kim M.K., Seo W.T., Lee Y.B., Cho K.M. (2013). Analyses of archaeal communities in *Doenjang* and *Ganjang* using a culture-independent manner based on 16S rRNA sequences. Food Sci. Biotechnol..

[10] Mun E., Park J.E., Cha Y. (2019). Effects of *Doenjang*, a traditional Korean soybean paste, with high-salt diet on blood pressure in Sprague–Dawley rats. Nutrients.

[11] Kim K.H., Lee S.H., Chun B.H., Jeong S.E., Jeon C.O. (2019). *Tetragenococcus halophilus* MJ4 as a starter culture for repressing biogenic amine (cadaverine) formation during saeu-jeot (salted shrimp) fermentation. Food Microbiol..

[12] Chun B.H., Kim K.H., Jeong S.E., Jeon C.O. (2020). The effect of salt concentrations on the fermentation of *doenjang*, a traditional Korean fermented soybean paste. Food Microbiol..

[13] Lee S., Oh D.-G., Lee S., Kim G.R., Lee J.S., Son Y.K., Bae C.-H., Yeo J., Lee C.H. (2015). Chemotaxonomic metabolite profiling of 62 indigenous plant species and its correlation with bioactivities. Molecules.

[14] Son S.Y., Kim N.K., Lee S., Singh D., Kim G.R., Lee J.S., Yang H.-S., Yeo J., Lee S., Lee C.H. (2016). Metabolite fingerprinting, pathway analyses, and bioactivity correlations for plant species belonging to the Cornaceae, Fabaceae, and Rosaceae families. Plant Cell Rep..

[15] Lee S.Y., Lee S., Lee S., Oh J.Y., Jeon E.J., Ryu H.S., Lee C.H. (2014). Primary and secondary metabolite profiling of *doenjang*, a fermented soybean paste during industrial processing. Food Chem..

[16] Lee S.Y., Kim H.Y., Lee S., Lee J.M., Muthaiya M.J., Kim B.S., Oh J.Y., Song C.K., Jeon E.J., Ryu H.S. (2012). Mass spectrometry-based metabolite profiling and bacterial diversity characterization of Korean traditional *meju* during fermentation. J. Microbiol. Biotechnol..

[17] Namgung H.J., Park H.J., Cho I.H., Choi H.K., Kwon D.Y., Shim S.M., Kim Y.S. (2010). Metabolite profiling of *doenjang*, fermented soybean paste, during fermentation. J. Sci. Food Agric..

[18] Nelson G., Chandrashekar J., Hoon M.A., Feng L., Zhao G., Ryba N.J., Zuker C.S. (2002). An amino-acid taste receptor. Nature.

[19] Jeong S.W., Kwon D.J., Gu M.S., Kim Y.S. (1994). Quality characteristics and preference of *Doenjang* using rice. Agric. Chem. Biotechnol..

[20] Munro I.C., Harwood M., Hlywka J.J., Stephen A.M., Doull J., Flamm W.G., Adlercreutz H. (2003). Soy isoflavones: A safety review. Nutr. Rev..

[21] Rostagno M.A., Villares A., Guillamón E., García-Lafuente A., Martínez J. (2009). Sample preparation for the analysis of isoflavones from soybeans and soy foods. J. Chromatogr. A.

[22] Lee S., Seo M.-H., Oh D.-K., Lee C.H. (2014). Targeted metabolomics for Aspergillus oryzae-mediated biotransformation of soybean isoflavones, showing variations in primary metabolites. Biosci. Biotechnol. Biochem..

[23] Rupasinghe H.V., Jackson C.-J.C., Poysa V., Di Berardo C., Bewley J.D., Jenkinson J. (2003). Soyasapogenol A and B distribution in soybean (*Glycine max* L. Merr.) in relation to seed physiology, genetic variability, and growing location. J. Agric. Food Chem..

[24] Jung J.Y., Chun B.H., Jeon C.O. (2015). Chromohalobacter is a causing agent for the production of organic acids and putrescine during fermentation of *ganjang*, a Korean traditional soy sauce. J. Food Sci..

[25] Shukla S., Park H.-K., Kim J.-K., Kim M. (2010). Determination of biogenic amines in Korean traditional fermented soybean paste (*Doenjang*). Food Chem. Toxicol..

[26] Chang M., Chang H.C. (2012). Development of a screening method for biogenic amine producing Bacillus spp.. Int. J. Food Microbiol..

[27] Han D.M., Chun B.H., Feng T., Kim H.M., Jeon C.O. (2020). Dynamics of microbial communities and metabolites in *ganjang*, a traditional Korean fermented soy sauce, during fermentation. Food Microbiol..

[28] Pannala A.S., Chan T.S., O’Brien P.J., Rice-Evans C.A. (2001). Flavonoid Bring chemistry and antioxidant activity: Fast reaction kinetics. Biochem. Biophys. Res. Commun..

[29] Takaaki Y., Michikatsu S. (1999). Isolation and properties of β-glucosidase produced by *Debaryomyces hansenii* and its application in winemaking. Am. J. Enol. Vitic..

[30] Pyo Y.-H., Lee T.-C., Lee Y.-C. (2005). Enrichment of bioactive isoflavones in soymilk fermented with b-glucosidase-producing lactic acid bacteria. Food Res. Int..

[31] Jeong D.-W., Kim H.-R., Jung G., Han S., Kim C.-T., Lee J.-H. (2014). Bacterial community migration in the ripening of *doenjang*, a traditional Korean fermented soybean food. J. Microbiol. Biotechnol..

[32] Coda R., Rizzello C.G., Di Cagno R., Trani A., Cardinali G., Gobbetti M. (2013). Antifungal activity of *Meyerozyma guilliermondii*: Identification of active compounds synthesized during dough fermentation and their effect on long-term storage of wheat bread. Food Microbiol..

[33] Xu X., Wu B., Zhao W., Lao F., Chen F., Liao X., Wu J. (2020). Shifts in autochthonous microbial diversity and volatile metabolites during the fermentation of chili pepper (*Capsicum frutescens* L.). Food Chem..

[34] Shim J.M., Lee K.W., Yao Z., Kim H.-J., Kim J.H. (2016). Properties of *doenjang* (soybean paste) prepared with different types of salts. J. Microbiol. Biotechnol..

[35] Kim T.-W., Lee J.-H., Kim S.-E., Park M.-H., Chang H.C., Kim H.-Y. (2009). Analysis of microbial communities in *doenjang*, a Korean fermented soybean paste, using nested PCR-denaturing gradient gel electrophoresis. Int. J. Food Microbiol..

