# Supplementary material for: Comprehensive Metabolite Profiling and Microbial Communities of Doenjang (Fermented Soy Paste) and Ganjang (Fermented Soy Sauce): A Comparative Study
Source: Foods. 2021 Mar 18;10(3):641. doi: 10.3390/foods10030641 (PMC8003076; doi:10.3390/foods10030641)
Supplement: Supplementary file 1 [file foods-10-00641-s001.pdf]

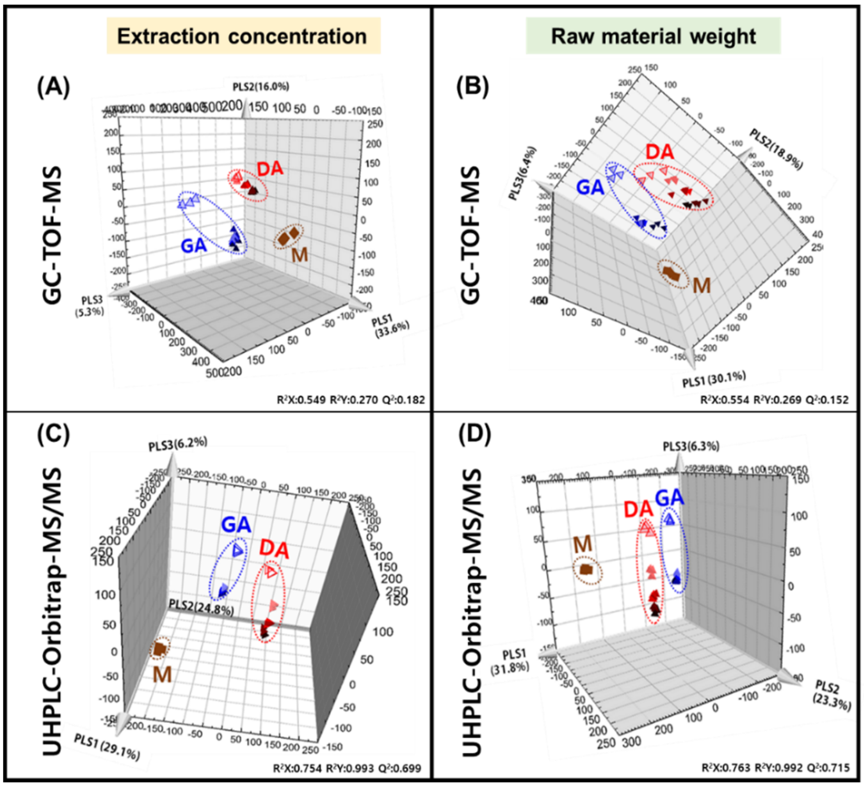

Figure S1. PLS-DA score plots based on concentrations derived from GC–TOF–MS (**A**) and UHPLC–Orbitrap–MS/MS (**C**). PLS-DA score plots normalized to raw material weight derived from GC–TOF–MS (**B**) and UHPLC–Orbitrap–MS/MS (**D**). Different processes symbolized as: *meju* (raw material: ◆), *doenjang* aging (0 d: ▲; 60 d: ▲; 90 d: ▲; 120 d: ▲; 360 d: ▲), *ganjang* aging (0 d: ▲; 60 d: ▲; 90 d: ▲; 120 d: ▲; 190 d: ▲).

.

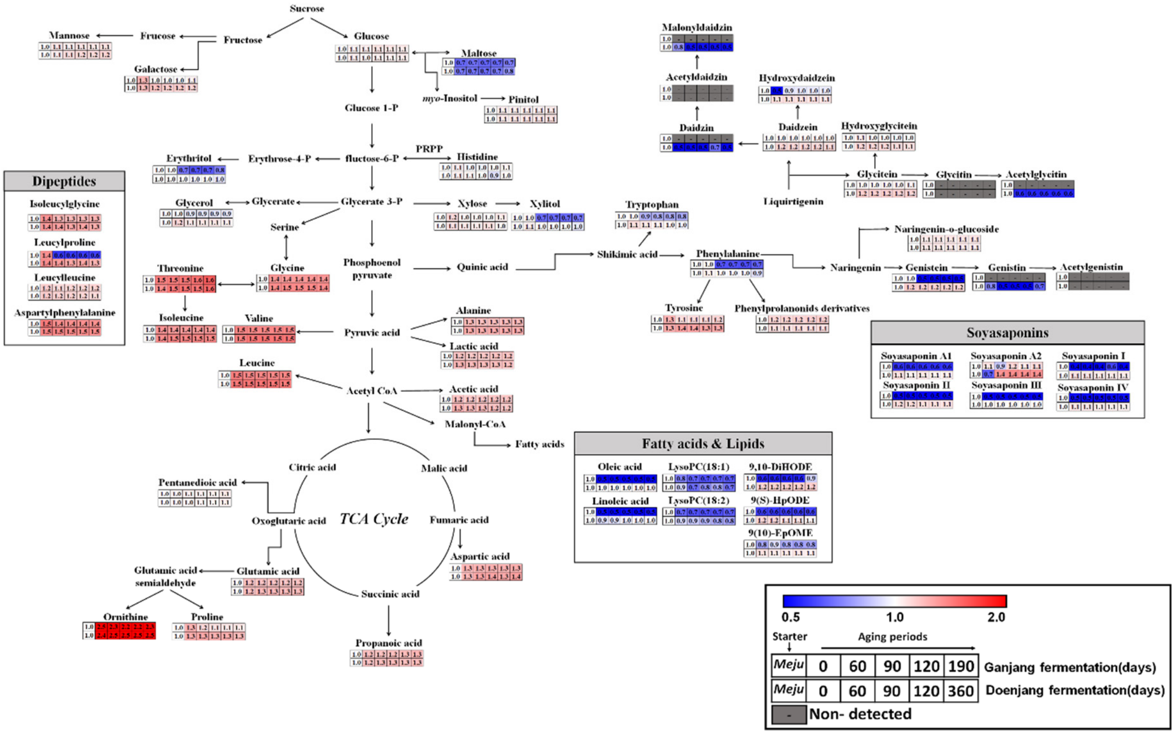

Figure. S2. Pathways of the relative levels of discriminant metabolites at different times of *doenjang* and *ganjang* as determined using the PLS-DA data sets based on raw material weight (VIP > 1.0) for GC–TOF–MS and UHPLC–Orbitrap–MS/MS analyses. The discriminant metabolites were further correlated with corresponding steps in the biosynthetic pathways adapted from the Kyoto Encyclopedia of Genes and Genomes database. The values indicate the log10-transformed fold changes based on the values of *meju*.

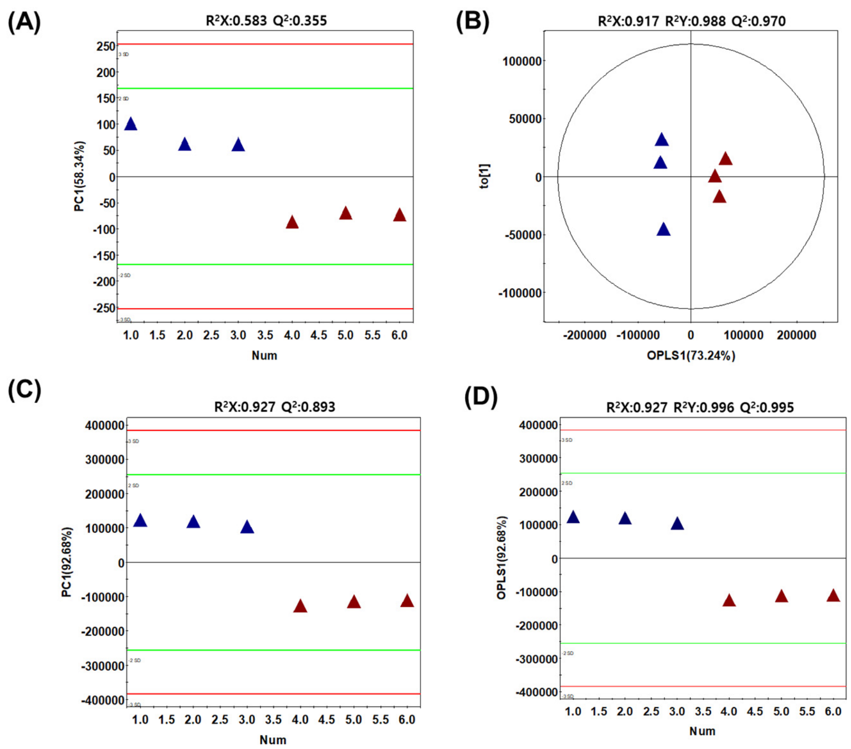

Figure. S3. PCA and OPLS-DA score plots derived from non-targeted metabolite profiling of *doenjang* and *ganjang* end products analyzed using GC–TOF-MS (**A**, **B**) and UHPLC–Orbitrap–MS/MS (**C**, **D**) (VIP > 1.5, $p < 0.05$). The score plot color codes indicate *doenjang* 360 d (▲) and *ganjang* 190 d (▲).

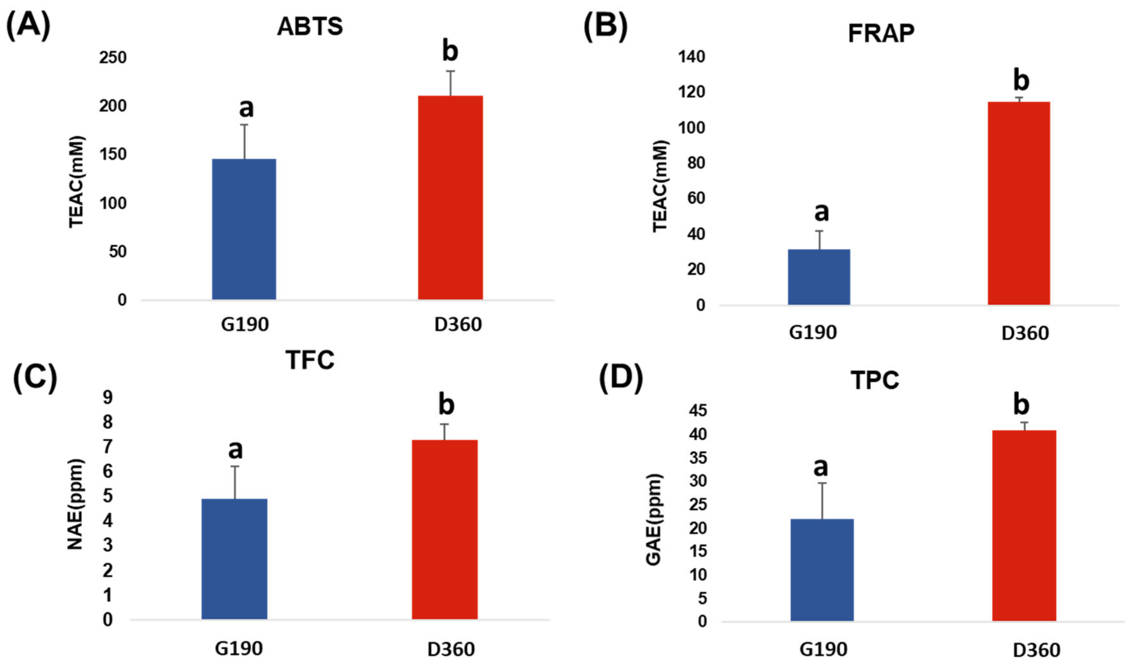

Figure. S4. Antioxidant activity analysis using ABTS (**A**), FRAP (**B**), TPC (**C**), and TFC (**D**) of *doenjang* (D360), and *ganjang* (G190) end-products. Different letters in the bar graph indicate significant differences as analyzed by ANOVA followed by Duncan's new multiple range test.

Table S1. Tentatively identified *meju* metabolites from different materials based on the GC-TOF-MS analysis.

NO.	Tentative identification[a]	GC-TOF-MS			
		RT(min)[b]	Identified *ion (m/z)*	Mass Fragment pattern	TMS[c]
	Amino acids				
1	Alanine	5.5	116	190,147,133,116,103,73,66	2
2	Valine	6.7	218	218,144,133,117,100,86,73,59	2
3	Leucine	7.2	102	158,138,102,75,73	2
4	Isoleucine	7.5	158	218,117,75,147,45,158,73	3
5	Proline	7.5	142	216,142,133,100,73,59	2
6	Glycine	7.6	174	248,188,174,158,133,117,100,86,73	3
7	Threonine	8.3	219	291,219,203,159,129,117,101,86	3
8	Aspartic acid	9.5	232	232,218,202,188,174	3
9	Glutamic acid	10.3	128	230,204,174,147,128,114,100,84,73	3
10	Ornithine	11.3	186	216,186,174,159,142,130,116,100,86	4
11	Tyrosine	12.6	100	147,133,100,73,59	3
12	Phenylalanine	10.4	218	218,192,177,160,147,120,100,91	2
13	Histidine	13.8	154	73,203,154,147,103,75,74,59	3
14	Tryptophan	14.3	202	348,231,202,174,130,95,73,45	3
	Fatty acids				
15	Linoleic acid	14.3	131	233,219,205,190,147	2
16	Oleic acid	14.2	117	313,201,117,75,73	1
	Organic acids				
17	Lactic acid	5.0	117	147,133,117,101,88,73,66	2
18	Acetic acid	5.1	66	177,147,133,117,103,81,73,66	2
19	Propanoic acid	6.1	147	233,218,177,147,130	2
20	Pentanedioic acid	9.9	129	147,129,116,103,101,85,75	3
	Sugar&Sugar derivatives				
21	Glycerol	7.3	117	147,133,117,103,89,73,59,55	3
22	Erythritol	9.4	217	307,277,217,189,147,103,73	4
23	Xylose	10.7	103	233,217,204,189,160	4
24	Xylitol	11.1	217	217,205,189,157,147	5
25	Maltose	16.7	361	174,130,100,86,73,59	8
26	Galactose	11.3	117	160,128,117,89,73,58	4
27	Mannose	12.3	319	160,147,129,117,103,89,73,59	5
28	Glucose	12.4	160	160,147,129,117,103,89	5
29	Pinitol	11.9	260	217,207,191,177,159	5
	Non-Identifications				
30	N.I 1	7.0	179	179,135,105,77,51	1
31	N.I 2	7.2	174	174,147,100,73,45,50	3
32	N.I 3	7.9	99	147,126,113,99,85,73,56	2
33	N.I 4	9.7	263	263,247,207,175,115,91	0
34	N.I 5	10.3	174	228,200,174,147,129,116,100,82,73	3
35	N.I 6	12.6	319	319,217,189,147,103,73,59	5
36	N.I 7	12.7	217	319,217,147,103,73,59	6

* The differential metabolites were selected using the VIP (>1.0) and *p*-values (<0.7) from the partial least squares-discriminant analysis model in Figure 2A. [a]: Retention time; [c]: TMS, trimethylsilyl; [a]: confirmed with the National Institutes of Standards and Technology (NIST) database and in-house libraries; STD, mass spectrum, consistent with that of the standard compounds.

Table S2. Tentatively identified *meju* metabolites from different materials based on the UHPLC-Orbitrap-MS/MS analysis.

NO.	Tentative identification	UHPLC-Orbitrap-MS/MS							
		RT(min)[a]	$[M-H]^-$	$[M+H]^+$	M.W.[b]	M.F.[c] $[M-H]^+$	Error (ppm)	MS fragmentation	ID
	Dipeptides								
37	Isoleucylglycine	1.32	187.1114	189.1224	188	C8 H17 O3 N2	-5.49	196,171,143>125,86	GNPS[d]
38	Leucylproline	1.94	227.1074	229.1534	228	C11 H21 O3 N2	-5.49	211,183,155,116>78,70	GNPS[d]
39	Leucylleucine	4.09	243.1745	245.1847	244	C12 H25 O3 N2	-5.10	235,227,199>69,57	GNPS[d]
40	Aspartylphenylalanine	2.36	279.1000	281.1144	280	C13 H17 O5 N2	-5.12	261>217,175,147,83	HMDB[e]
	Biogenic amines								
41	Phenylethylamine	1.44	-	122.0971	121	C8 H12 N	-5.70	119,112,105>79,76,68	STD
42	Histamine	0.65	-	112.0875	111	C5 H10 N3	-2.98	108,97,95>68	STD
43	Tryptamine	3.02	-	161.1080	160	C10 H12 N2	-7.36	-	STD
	Phenylpropanoids								
44	Phenylpropanoid derivatives	3.36	293.1187	295.1306	294	C19 H17 O3 [-]	1.34	275,221,187,>164,127(-)	HMDB[e]
	Flanvonoids								
45	Malonyldaidzin	4.92	-	503.1198	502	C24 H23 O12	-1.93	457,344,253>224,209,181(-)	Ref [1]
46	Acetyldaidzin	5.26	457.1139	459.1275	458	C23 H23 O10	-2.27	441,255>227,199,137	Ref [1]
47	Acetylglycitin	5.30	487.1245	489.1379	488	C24 H25 O11	-2.57	-	Ref [1]
48	Acetylgenistin	5.71	473.1080	475.1223	474	C23 H23 O11	-2.42	457,271>243,215,153	Ref [1]
49	Daidzin	4.51	415.1034	417.1171	416	C21 H21 O9	-2.04	287,255>227,199,137	Ref [1]
50	Glycitin	4.61	445.1133	447.1274	446	C22 H23 O10	-2.60	429,285>270,229,167	Ref [1]
51	Genistin	4.92	431.0977	433.1140	432	C21 H21 O10	-1.99	433>271,253,243,215,153	Ref [1]
52	Daidzein	5.67	253.0524	255.0676	254	C15 H11 O4	4.50	255,227,199>181,170,157	Ref [1]
53	Glycitein	5.78	283.0644	285.0786	284	C16 H13 O5	-5.47	270,253,229,166>152,108	Ref [1]
54	Genistein	6.31	269.0500	271.0625	270	C15 H11 O5	4.70	253,243158,152>147,130	Ref [1]
55	Hydroxydaidzein	4.89	269.0492	271.0600	270	C15 H11 O5	-5.20	271,253,243,225>152,132	Ref [2]
56	Hydroxyglycitein	5.05	299.0598	301.0692	300	C16 H13 O6	-4.83	301,286,245,213>167,139	Ref [2]
57	Naringenin-O-glucoside	3.62	433.1181	435.1332	434	C21H23O10	-4.28	433> 415, 343, 313> 285, 269	Ref [3]
	Soyasaponins								
58	Soyasaponin A1	5.08	1267.6111	1269.6049	1269	C59 H97 O29	-4.84	1107,945,747,615>351,203	Ref [4]
59	Soyasaponin A2	5.31	1105.5488	1107.5699	1107	C53 H87 O24	-6.24	945,813,729,597,579>403,363,253	Ref [4]
60	Soyasaponin I	6.99	941.5245	943.5392	943	C48 H77 O18P [-]	4.73	925,797,617,581,551>423,405,351	Ref [5]
61	Soyasaponin II	7.19	911.5071	913.5102	913	C47 H77 O17	-5.84	893,615,525,457>437,409,393,233(-)	Ref [5]
62	Soyasaponin III	7.22	795.4650	797.4760	797	C42 H69 O14	-6.71	777,759,613>467,413(-)	Ref [5]
63	Soyasaponin IV	7.32	765.4499	767.4536	766	C41 H67 O13	-5.30	615,457,409,357>295(-)	Ref [5]
64	Soyasapogenol A	9.75	-	457.3678 [f]	475	C30 H49 O3	6.26	439,421,403>393	STD
65	Soyasapogenol B	10.06	-	441.3697 [f]	458	C30 H49 O2	6.02	423,405>283,203,187	STD
	Lipids & Fatty acids								
66	LysoPC(18:2)	8.39	518.2715	520.3448	519	C26 H51 O7 N P	-5.83	520> 502> 443> 184	Ref [6]
67	LysoPC(18:0)	9.52	568.3691 [g]	524.3757	523	C26 H55 O7 N P	-5.41	524> 506, 447> 311, 184	Ref [6]
68	9,10-DiHODE	7.11	311.2258	313.2370	312	C18 H33 O4	-4.57	275,249>231,177,127	Ref [7]
69	9(S)-HpODE	7.60	311.2256	313.2359	312	C18 H33 O4	-5.66	275,249>231,177,97	Ref [7]
70	9(10)-EpOME	9.56	295.2313	297.2408	296	C18 H33 O3	-9.11	277,249>155,141,127	GNPS[d]
	Non-identification								
71	N.I. 7	10.27	390.3045	392.3195	391	-	-	391,374,346,261,243>241,212	-
72	N.I. 8	7.83	374.2517	376.2627	375	-	-	358,343,302,293>218,135,107	-
73	N.I. 9	10.47	378.3048	380.3195	379	-	-	379,362,334>243,212,170	-

* The differential metabolites were selected using the VIP (>1.0) and *p*-values (<0.7) from the partial least squares discriminant analysis model in Figure 3A. [a]: RT, retention time; [b]: M.W., molecular weight; [c]: M.F., molecular formula; [d] :GNPS, https://gnps.ucsd.edu/; [e]: HMDB, https://hmdb.ca/; [f]: $[M-H_2O]^+$; [g]: $[M-FA+H]^-$.

Table S3. Bacterial and fungal illumina data sets derived from *meju*, *doenjang*, and *ganjang* samples and their statistical diversity analysis.

Sample name	Time (day)	Bacteria[a]				Fungi[a]			
		High quality reads	OTUs	Chao1	Shannone-Weaver	High quality reads	OTUs	Chao1	Shannone-Weaver
Meju	–	9,773	33	36.9	3.3	127,781	15	15.6	0.1
Doenjang	0	1,450	66	75.1	4.6	34,709	16	16	2.4
	60	14,243	69	98.0	4.2	43,934	18	18	2.8
	90	11,645	69	90.5	4.3	36,376	13	13	1.4
	120	7,738	59	81.4	4.1	42,927	17	17	2.1
	360	944	37	37.9	3.6	50,337	28	28	2.7
Ganjang	0	13,583	66	89.0	4.4	32,272	15	15	2.4
	60	4,866	58	67.5	4.0	95,935	17	16.2	1.2
	90	5,421	50	56.5	4.0	37,723	29	29	2.4
	120	6,875	46	53.6	3.9	101,559	43	42.7	2.0
	190	9,174	44	58.9	3.8	85,438	32	32.1	2.0

Abbreviation: OTU, operational taxonomic unit.

[a] The bacterial and fungal sequences in each *doenjang* samples were normalized to 944 and 32,272, respectively and diversity indices in each *doenjang* samples were calculated using the normalized sequences.

Supplementary references

1. Lee, S.Y.; Lee, S.; Lee, S.; Oh, J.Y.; Jeon, E.J.; Ryu, H.S.; Lee, C.H. Primary and secondary metabolite profiling of doenjang, a fermented soybean paste during industrial processing. *Food Chem.* **2014**, *165*, 157-166.
2. Lee, S.; Seo, M.-H.; Oh, D.-K.; Lee, C.H. Targeted metabolomics for Aspergillus oryzae-mediated biotransformation of soybean isoflavones, showing variations in primary metabolites. *Biosci. Biotechnol. Biochem.* **2014**, *78*, 167-174.
3. Zeng, X.; Su, W.; Zheng, Y.; Liu, H.; Li, P.; Zhang, W.; Liang, Y.; Bai, Y.; Peng, W.; Yao, H. UFLC-Q-TOF-MS/MS-based screening and identification of flavonoids and derived metabolites in human urine after oral administration of Exocarpium Citri Grandis extract. *Molecules.* **2018**, *23*, 895.
4. Suh, D.H.; Jung, E.S.; Park, H.M.; Kim, S.H.; Lee, S.; Jo, Y.H.; Lee, M.K.; Jung, G.; Do, S.-G.; Lee, C.H. Comparison of metabolites variation and antiobesity effects of fermented versus nonfermented mixtures of Cudrania tricuspidata, Lonicera caerulea, and soybean according to fermentation in vitro and in vivo. *PloS one.* **2016**, *11*.
5. Lee, S.-Y.; Kim, J.-S.; Shim, S.-H.; Kang, S.-S. Soyasaponins from Soybean Flour Medium for the Liquid Culture of Ganoderma applanatum. *B. Korean. Chem. Soc.* **2011**, *32*, 3650-3654.
6. Kwon, Y.S.; Lee, S.; Lee, S.H.; Kim, H.J.; Lee, C.H. Comparative Evaluation of Six Traditional Fermented Soybean Products in East Asia: A Metabolomics Approach. *Metabolites.* **2019**, *9*, 183.
7. Strassburg, K.; Huijbrechts, A.M.; Kortekaas, K.A.; Lindeman, J.H.; Pedersen, T.L.; Dane, A.; Berger, R.; Brenkman, A.; Hankemeier, T.; van Duynhoven, J. Quantitative profiling of oxylipins through comprehensive LC-MS/MS analysis: application in cardiac surgery. *Anal. Bioanal. Chem.* **2012**, *404*, 1413-1426.
